# Disparities in the first-ever diagnosed liver cancers between the emergency department and outpatient department in Taiwan: a population-based study

**DOI:** 10.1186/s12889-023-15218-5

**Published:** 2023-02-08

**Authors:** Tai-Yi Hsu, Jhu-Jing Ye, Sih-Yun Ye, Hsiao-Yuan Tseng, Wen-Yu Chou, Pei-Tseng Kung, Wen-Chen Tsai

**Affiliations:** 1 Department of Emergency Medicine, China Medical University & Hospital, Taichung, Taiwan R.O.C.; 2 Department of Public Health, China Medical University, Taichung, Taiwan R.O.C.; 3 Department of Health Services Administration, China Medical University, 100, Sec. 1, Jingmao Rd., Beitun Dist, 406040 Taichung, Taiwan R.O.C.; 4 Department of Medical Research, China Medical University Hospital, China Medical University, Taichung, Taiwan R.O.C.; 5 Department of Healthcare Administration, Asia University, Taichung, Taiwan R.O.C.

**Keywords:** Liver cancer, Emergency department, Survival analysis, Health examination

## Abstract

**Background:**

Liver cancer is ranked fifth in incidence and second in mortality among cancers in Taiwan. Nevertheless, the Taiwan government does not screen for liver cancer in its free cancer screening and preventive health examination service. This study compared the differences in cancer stage and survival between patients who received an initial liver cancer diagnosis in outpatient departments (OPDs) and those who received such a diagnosis in emergency departments (EDs).

**Methods:**

This retrospective cohort study used the 2000–2016 National Health Insurance Database to obtain a sample from 2 million Taiwanese residents. To evaluate the effect of the utilization of the adult health examination offered to people aged ≥ 40 years, patients aged ≥ 40 years who received an initial liver cancer diagnosis between 2003 and 2015 were followed up until December 31, 2016.

**Results:**

In total, 2,881 patients were included in this study. A greater proportion of cancer cases in the OPD group were non-advanced than those in the ED group (75.26% vs. 54.23%). Having stage C or D cancer, having a low monthly salary, and a Charlson comorbidity index score ≥ 8, not having hepatitis B, being divorced, and attending a non-public hospital as the primary care institution were risk factors for initial ED diagnosis. The risk of liver cancer-specific death among the ED group patients was 1.38 times that among the OPD group patients (adjusted hazard ratio = 1.38, 95% confidence interval [CI] = 1.14–1.68, P < 0.001). However, the use of health examination did not exert a significant effect on the likelihood of liver cancer diagnosis in an ED (adjusted odds ratio = 0.86, 95% CI = 0.61–1.21, P = 0.381).

**Conclusion:**

Government-subsidized health examinations are insufficient to prevent first-ever diagnosed liver cancers in EDs. Patients with liver cancers diagnosed in EDs had a higher risk of advanced stage and mortality. For early detection and treatment, the government may consider implementing liver cancer screening for high-risk and low-socioeconomic people.

## Background

Liver cancer is the second leading cause of cancer-related death in men and the sixth most common cancer worldwide, with 830,180 deaths and an increasing incidence of over 900,000 new cases recorded in 2020 [1]. Liver cancer is prevalent specifically in Taiwan and Eastern Asia. Taiwan reported 10,988 new cases in 2020 [2], which equates to nearly 30 people receiving a diagnosis of liver cancer daily. Taiwanese government statistics suggest that among the 51,656 people who died of malignancy in 2021, 7,970 died of liver cancer [3]. Only lung cancer caused more deaths. From 1984 to 1998, liver cancer was the deadliest cancer in Taiwan, eventually surpassed by lung cancer in 1999 [4, 5].

Liver cancer is frequently diagnosed late in its course because of the absence of symptoms in patients with early-staged cancer. Only regular screening and follow-up in outpatient departments (OPDs) can enable the early diagnosis of liver cancer [6, 7]. If liver cancer is discovered during an emergency department (ED) visit, the symptoms or complications are typically severe [8]. Currently, Taiwan uses the Barcelona Clinic Liver Cancer (BCLC) classification as the standard staging system, which divides the disease into stages 0, A, B, C, and D [9]. The liver cancer stage is a factor affecting survival [10]. According to a cancer care report from a Taiwanese medical center, the 5-year relative survival rate of patients with liver cancer at stages 0 to D was 74%, 70.9%, 36.5%, 16.6%, and 11.2%, respectively [11]. In addition, the average cost of drugs for liver cancer treatment was NT$59,780 and the average medical cost per patient was NT$162,276 per person in 2018 [12]. When considering 5-year survival as a case of successful treatment, sustaining each patient costs an average of NT$2,273,554 [13].

Several studies have confirmed that hepatitis B and C are the main cause of liver cancer in Taiwan [14, 15]. A survey of individuals with hepatitis revealed that 85.9% of patients undertaking regular self-paid liver cancer screenings and follow-ups were in BCLC stages 0 and A at the time of diagnosis, whereas only 39% of the people who did not follow up regularly were in the early disease stages (0 or A) [16]. This demonstrates that regular screening and follow-up can effectively detect liver cancer earlier. However, the cancer screening and adult health examination services currently subsidized by the Taiwan government do not include liver cancer, and free hepatitis B and C screenings are only provided once in a lifetime for qualified persons [17].

Accordingly, studies have noted that regular OPD-based screenings can detect liver cancer at an earlier stage. However, no study has investigated the difference between the cancer stage and survival of patients with liver cancer who received an initial diagnosis in an OPD versus those who received such diagnosis in an ED in Taiwan to explore the effect of current adult health examination policy on the prevention of liver cancer.

## Methods

### Data sources

In this research, a retrospective cohort study design was adopted. The data for this study were obtained from the 2 million representative samples recorded in the 2000–2016 National Health Insurance Research Database (NHIRD), which was used to enroll study participants. We also linked this data with that of the Taiwan Cancer Registry from 2003 to 2015 and the Ministry of Health and Welfare’s Cause of Death File from 2003 to 2016. The Taiwan Cancer Registry contained information on cancer cases as well as relevant information such as patients’ cancer stage and time of cancer diagnosis. Cancer was diagnosed in accordance with the International Classification of Diseases (ICD) for Oncology, third edition (ICD-O-3), which identifies cancer categories according to the primary site, histology, behavioral code, and classification or differentiation. In determining the liver cancer stage according to the diagnostic results, the Taiwan Cancer Registry recorded data on the clinical, surgical, and pathological severity of cancers in accordance with the BCLC staging system.

### Study population

**Fig. 1:**
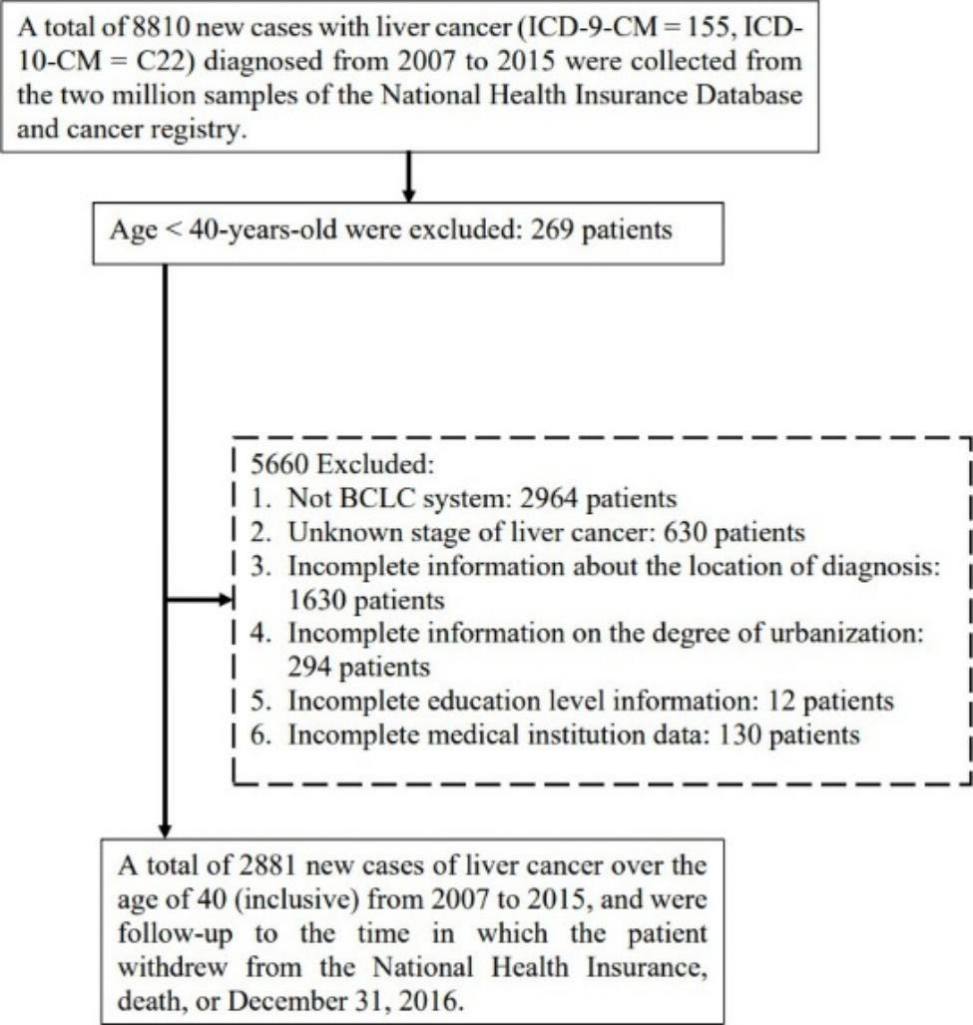
Flowchart of the selection of the study participants

In this study, patients who received an initial diagnosis of liver cancer (ICD, 9th revision, Clinical Modification code 155, 10th revision of ICD and ICD-O-3 codes C22.0–C22.1) between 2003 and 2015 were selected as the study participants and were followed up until December 31, 2016. To evaluate the effect of utilization of the preventive health examination offered to Taiwanese adults over 40 years old, only patients aged over 40 years were enrolled in the study. The participants were retrospectively tracked from 2000 onwards to determine whether they received adult health examinations within 3 years of receiving an initial liver cancer diagnosis. The last visit date with relevant diagnosis and treatment for liver cancer before the cancer registration was acquired to determine the location of diagnosis (i.e. OPD or ED). Patients with the following conditions were excluded: liver cancer staging based on a system other than the BCLC, unknown liver cancer stage, incomplete details of the location of diagnosis, incomplete details of residential area (urbanization), incomplete details of education level, and incomplete medical institution data (Fig. 1).

### Definition of relevant variables

The study variables were as follows: location of first-ever liver cancer diagnosis (OPD: 2,704 patients, ED: 177 patients); patient characteristics (e.g. sex, age, and marital status [unmarried, married, divorced, or widowed]); monthly salary (≤ NT$22,800, 22,801–28,800, or > 28,800); education level (elementary school or lower, junior high school, senior high school or higher); urbanization of residence areas (7 levels, with level 1 being the most urbanized) [18]; level of preventive health utilization (completed adult health exam during the 3 years before liver cancer diagnosis); level of health care organization (medical center, regional hospital, district hospital, or primary clinic); ownership status of the medical organization (public or non-public); and health status in terms of Charlson comorbidity index (CCI) score and hepatitis B and C diagnoses. CCI score was determined based on the patients’ primary and secondary diagnosis codes either twice in the OPD or once during hospitalization 1 year prior to the diagnosis of liver cancer and assigned a score of ≤ 2, 3–7, or ≥ 8 [19]; Hepatitis B or C infection were determined based on whether the patient data included diagnosis codes for hepatitis B or C twice in the OPD or once during hospitalization 1 year prior to the diagnosis of liver cancer.

### Statistical methods

Descriptive data of the seven study variables, namely the location of diagnosis, personal characteristics, socioeconomic status, environmental factors, preventive health utilization, health status, and characteristics of the main hospital before diagnosis, are presented as numbers and percentages. The chi-squared test was used to assess whether the variables differed significantly between the OPD and ED patient groups. Multivariate logistic regression was performed to explore their correlation with the initial location of diagnosis and liver cancer stage (early or advanced). Log-rank test was performed to determine whether the survival of patients with liver cancer among patients differed significantly based on the location of diagnosis. Cox proportional hazard regression models were employed to estimate the adjusted hazard ratio (aHR) of death after controlling for confounders, including the location of diagnosis, personal characteristics, socioeconomic status, environmental factors, use of preventive health, health status, and characteristics of the primary care institutions. The follow-up duration was from the time of diagnosis of liver cancer to the time the patient withdrew from the NHI claims scheme, death, or December 31, 2016. The statistical analyses were performed using SAS (version 9.1; SAS Institute, Cary, NC, USA), and *P* values of < 0.05 were considered statistically significant. This study was approved by the research ethics committee of China Medical University and Hospital (Institutional Review Board No. CRREC-109-156).

## Results

In this study, analyses of descriptive statistics were initially conducted to determine the basic characteristics of the two patient groups (with liver cancer diagnoses from the OPD or ED). A total of 2,881 patients were enrolled in this study, of which 2,704 (93.86%) and 177 (6.14%) received an initial liver cancer diagnosis in an OPD and ED, respectively (Table 1). In terms of staging, the majority of diagnoses received in an OPD were of stage A liver cancer (42.53%), whereas those received in an ED were primarily of stage C (36.72%). In terms of sex, male patients outnumbered their female counterparts in both the OPD and ED groups. Male patients accounted for 70.67% and 73.45% of the OPD and ED group members, respectively. In both groups, the majority of the patients were aged 55–64 years (30.88% and 32.20% in the OPD and ED groups, respectively). The chi-squared test results revealed that the following variables differed significantly (*P* < 0.05) between the OPD and ED groups: cancer stage, age at diagnosis, marital status, monthly salary, health examination in the 3 years prior to diagnosis, severity of comorbidities, hepatitis B infection, and the post-diagnosis medical institution level.

**Table 1:** Variables associated with the location of diagnosis of first-ever liver cancer

Variables	Total		Outpatient department		Emergency department		P-value^a^	Adjusted OR^b^	95%CI^c^		P-value^d^
	N	%	N	%	N	%					
**Total**	2881	100.00	2704	93.86	177	6.14					
**Stages**							< 0.001				
0	216	7.50	212	7.84	4	2.26		1.00			
A	1191	41.34	1150	42.53	41	23.16		1.61	0.56	4.60	0.373
B	724	25.13	673	24.89	51	28.81		2.81	0.98	8.01	0.054
C	652	22.63	587	21.71	65	36.72		3.40	1.19	9.68	0.022*
D	98	3.40	82	3.03	16	9.04		5.36	1.68	17.14	0.005*
**Personal characteristics**											
**Gender**							0.483				
Male	2041	70.84	1911	70.67	130	73.45		1.00			
Female	840	29.16	793	29.33	47	26.55		0.89	0.60	1.31	0.551
Age (Year)							0.041				
40–54	547	18.99	510	18.86	37	20.90		1.00			
55–64	892	30.96	835	30.88	57	32.20		1.03	0.64	1.67	0.900
65–74	871	30.23	833	30.81	38	21.47		0.61	0.36	1.06	0.080
≥75	571	19.82	526	19.45	45	25.42		0.98	0.56	1.71	0.953
Mean ± SD	64.51 ± 10.86		64.52 ± 10.81		64.44 ± 11.62		0.922				
Marital status							0.009				
Unmarried	155	5.38	147	5.44	8	4.52		1.00			
Married	2356	81.78	2224	82.25	132	74.58		1.60	0.73	3.48	0.238
Divorced	144	5.00	128	4.73	16	9.04		2.97	1.18	7.50	0.021*
Widowed	226	7.84	205	7.58	21	11.86		2.50	1.00	6.28	0.051
Socioeconomic status											
**Monthly salaries (NT$** ^**e**^ **)**							< 0.001				
≤22,800	1791	62.17	1643	60.76	148	83.62		1.00			
22,801 − 28,800	660	22.91	645	23.85	15	8.47		0.40	0.22	0.70	0.002*
≥28,801	430	14.93	416	15.38	14	7.91		0.64	0.36	1.17	0.149
**Education level**							0.107				
Elementary school (and illiteracy)	1542	53.52	1449	53.59	93	52.54		1.00			
Junior high school	526	18.26	484	17.90	42	23.73		1.32	0.85	2.05	0.212
Senior high school (and above)	813	28.22	771	28.51	42	23.73		0.94	0.61	1.46	0.785
**Environmental factors**											
**Urbanization**							0.630				
Level 1	657	22.80	619	22.89	38	21.47		1.00			
Level 2	839	29.12	789	29.18	50	28.25		1.03	0.66	1.61	0.909
Level 3	444	15.41	409	15.13	35	19.77		1.43	0.87	2.34	0.162
Level 4	506	17.56	480	17.75	26	14.69		0.90	0.52	1.55	0.709
Level 5	103	3.58	96	3.55	7	3.95		1.37	0.57	3.30	0.487
Level 6	190	6.59	176	6.51	14	7.91		1.32	0.67	2.60	0.415
Level 7	142	4.93	135	4.99	7	3.95		0.76	0.32	1.79	0.527
**Use of preventive medicine**											
**Health examination within three years before the diagnosis**							< 0.001				
No	793	27.53	724	26.78	69	38.98		1.00			
Yes	2088	72.47	1980	73.22	108	61.02		0.86	0.61	1.21	0.381
**Health Status**											
**Charlson Comorbidity Index**							< 0.001				
≤ 2	375	13.02	366	13.54	9	5.08		1.00			
3–7	1907	66.19	1789	66.16	118	66.67		2.02	0.99	4.11	0.052
≥8	599	20.79	549	20.30	50	28.25		2.17	1.02	4.63	0.044*
Hepatitis B							< 0.001				
Yes	1113	38.63	1066	39.42	47	26.55		1.00			
No	1768	61.37	1638	60.58	130	73.45		1.61	1.11	2.33	0.011*
Hepatitis C							0.394				
No	1843	63.97	1724	63.76	119	67.23		1.00			
Yes	1038	36.03	980	36.24	58	32.77		0.91	0.64	1.31	0.619
**Characteristics of the main hospital before diagnosis**											
**Level of medical institution**							0.274				
Medical center	317	11.00	292	10.80	25	14.12		1.00			
Regional hospital	527	18.29	497	18.38	30	16.95		0.62	0.35	1.11	0.110
District hospital	310	10.76	286	10.58	24	13.56		0.94	0.49	1.78	0.838
Primary clinic	1727	59.94	1629	60.24	98	55.37		0.57	0.33	1.00	0.051
**Ownership of medical institutions**							0.467				
Public hospital	675	23.43	638	23.59	37	20.90		1.00			
Non-public hospital	2206	76.57	2066	76.41	140	79.10		1.62	1.01	2.58	0.044*
**Characteristics of main treatment institutions after diagnosis**											
**Level of medical institution**							0.003				
Medical center	494	17.15	449	16.61	45	25.42		--			
Regional hospital	793	27.53	746	27.59	47	26.55		--	--	--	--
District hospital	332	11.52	306	11.32	26	14.69		--	--	--	--
Primary clinic	1262	43.80	1203	44.49	59	33.33		--	--	--	--
**Ownership of medical institutions**							0.364				
Public hospital	444	15.41	412	15.24	32	18.08		--			
Non-public hospital	2437	84.59	2292	84.76	145	81.92		--	--	--	--

^a^Chi-squared test. ^b^Event is a diagnosis in an emergency department; OR: odds ratio. ^c^Confidence interval. ^d^Logistic regression analysis. ^e^NT$: New Taiwan dollar. *P < 0.05

The multivariate logistic regression results indicated that patients who received an initial liver cancer diagnosis in an ED were more likely to be at an advanced disease stage (stage C: adjusted odds ratio [aOR] = 3.40, 95% confidence interval [CI] = 1.19–9.68; Stage D: aOR = 5.36, 95% CI = 1.68–17.14), divorced (aOR = 2.97, 95% CI = 1.18–7.50), have lower monthly salary, CCI score of ≥ 8 (aOR = 2.17, 95% CI = 1.02–4.63), not have hepatitis B (aOR = 1.61, 95% CI = 1.11–2.33), and in a non-public hospital (aOR = 1.62, 95% CI = 1.01–2.58). However, the use of health examination did not exert a significant effect on the likelihood of liver cancer diagnosis in an ED (adjusted odds ratio = 0.86, 95% CI = 0.61–1.21, *P* = 0.381; Table 1).

In this study, the impact of each variable on the stage of liver cancer was further analyzed. Table 2 indicates that compared with liver cancer cases initially diagnosed in OPDs, those initially diagnosed in EDs had higher proportions of stages B (28.81% vs. 24.89%), C (36.72% vs. 21.71%), and D (9.04% vs. 3.03%); by contrast, the proportions of stage 0 (2.26% vs. 7.84%) and A (23.16% vs. 42.53%) liver cancer cases diagnosed in an ED were lower than those of cases diagnosed in an OPD (*P* < 0.05).

**Table 2:** The association between the stages of first-ever diagnosed liver cancer and the related variables

Variables	Stage 0		Stage A		Stage B		Stage C		Stage D		P-value^a^
	N	%	N	%	N	%	N	%	N	%	
**Total**	216	7.50	1191	41.34	724	25.13	652	22.63	98	3.40	
**Location of diagnosis**											< 0.001
Outpatient department	212	7.84	1150	42.53	673	24.89	587	21.71	82	3.03	
Emergency department	4	2.26	41	23.16	51	28.81	65	36.72	16	9.04	
**Personal characteristics**											
**Sex**											< 0.001
Male	137	6.71	805	39.44	559	27.39	474	23.22	66	3.23	
Female	79	9.40	386	45.95	165	19.64	178	21.19	32	3.81	
**Age (Year)**											< 0.001
40–54	44	8.04	209	38.21	117	21.39	158	28.88	19	3.47	
55–64	67	7.51	409	45.85	199	22.31	196	21.97	21	2.35	
65–74	68	7.81	366	42.02	243	27.90	169	19.40	25	2.87	
≥75	37	6.48	207	36.25	165	28.90	129	22.59	33	5.78	
**Marital status**											< 0.001
Unmarried	10	6.45	55	35.48	28	18.06	52	33.55	10	6.45	
Married	188	7.98	984	41.77	605	25.68	512	21.73	67	2.84	
Divorced	5	3.47	67	46.53	29	20.14	37	25.69	6	4.17	
Widowed	13	5.75	85	37.61	62	27.43	51	22.57	15	6.64	
**Socioeconomic status**											
**Monthly salaries (NT$** ^**b**^ **)**											< 0.001
≤22,800	70	3.91	595	33.22	492	27.47	542	30.26	92	5.14	
22,801 − 28,800	86	13.03	346	52.42	164	24.85	61	9.24	3	0.45	
≥28,801	60	13.95	250	58.14	68	15.81	49	11.40	3	0.70	

^a^Chi-squared test. ^b^NT$: New Taiwan dollar

Table 3 details the results of the multivariate logistic regression used to explore the factors related to the probability of advanced-stage liver cancer among the study participants, revealing significant differences based on the location of initial diagnosis, age at diagnosis, monthly salary, education level, use of preventive health examination, CCI score, hepatitis B and C infections, and the ownership of the main hospital attended before diagnosis (*P* < 0.05). The aOR of advanced-staged liver cancer diagnosis was 1.77 times higher in the ED group than in the OPD group (95% CI = 1.27–2.47, *P* < 0.001). Therefore, receiving an initial liver cancer diagnosis in an ED entailed a higher probability of advanced-stage cancer than receiving a diagnosis in an OPD. In addition, patients who have received adult health examinations within the 3 years before diagnosis had a 0.76 times lower risk of advanced stage liver cancer (95% CI = 0.62–0.92, *P* = 0.005) compared with those who did not receive such an examination.

**Table 3:** Multivariate logistic regression analysis to explore the related variables for the probability of advanced liver cancers

Variables	Non-advanced*		Advanced*		P-value^a^	Adjusted OR^b^	95%CI^c^		P-value^d^
	N	%	N	%					
**Total**	2131	73.97	750	26.03					
**Location of diagnosis**					< 0.001				
Outpatient department	2035	75.26	669	24.74					
Emergency department	96	54.23	81	45.76		1.77	1.27	2.47	< 0.001*
**Personal characteristics**									
**Gender**					< 0.001				
Male	1501	73.54	540	26.45					
Female	630	74.99	210	25.00		1.06	0.85	1.32	0.605
**Age (Year)**					< 0.001				
40–54	370	67.64	177	32.35					
55–64	675	75.67	217	24.32		0.68	0.52	0.90	0.007*
65–74	677	77.73	194	22.27		0.54	0.40	0.73	< 0.0001*
≥75	409	71.63	162	28.37		0.64	0.46	0.88	0.007*
Marital Status					< 0.001				
Unmarried	93	59.99	62	40.00					
Married	1777	75.43	579	24.57		0.69	0.47	1.01	0.055
Divorced	101	70.14	43	29.86		0.76	0.45	1.28	0.296
Widowed	160	70.79	66	29.21		0.77	0.47	1.27	0.307
Socioeconomic status									
**Monthly salaries (NT$** ^**e**^ **)**					< 0.001				
≤22,800	1157	64.60	634	35.40					
22,801 − 28,800	596	90.30	64	9.69		0.25	0.18	0.33	< 0.0001*
≥28,801	378	87.90	52	12.10		0.31	0.23	0.43	< 0.0001*
**Education level**					0.120				
Elementary school (and illiteracy)	1131	73.34	411	26.65					
Junior high school	378	71.86	148	28.13		0.82	0.63	1.07	0.148
Senior high school (and above)	622	76.51	191	23.49		0.69	0.54	0.89	0.004*
**Environmental status**									
**Urbanization**					0.254				
Level 1	478	72.76	179	27.24					
Level 2	610	72.70	229	27.29		1.01	0.79	1.30	0.934
Level 3	331	74.54	113	25.45		0.90	0.67	1.21	0.481
Level 4	390	77.07	116	22.93		0.88	0.65	1.18	0.382
Level 5	84	81.56	19	18.44		0.66	0.37	1.17	0.154
Level 6	136	71.58	54	28.42		1.14	0.76	1.70	0.526
Level 7	102	71.82	40	28.17		1.09	0.70	1.70	0.708
**Use of preventive medicine**									
**Health examination within three years before the diagnosis**					< 0.001				
No	501	63.19	292	36.83					
Yes	1630	78.07	458	21.94		0.76	0.62	0.92	0.005*
**Health status**									
**Charlson comorbidity index**					< 0.001				
≤ 2	319	85.07	56	14.94					
3–7	1450	76.04	457	23.97		1.37	0.99	1.90	0.056
≥ 8	362	60.43	237	39.57		2.18	1.52	3.13	< 0.0001*
Hepatitis B					< 0.001				
No	1255	70.98	513	29.01					
Yes	876	78.71	237	21.30		0.61	0.50	0.75	< 0.0001*
Hepatitis C					< 0.001				
No	1291	70.05	552	29.95					
Yes	840	80.93	198	19.08		0.48	0.39	0.59	< 0.0001*
**Characteristics of the main hospital before diagnosis**									
**Level of the medical institutions**					0.529				
Medical center	227	71.62	90	28.39					
Regional hospital	401	76.09	126	23.90		0.73	0.51	1.03	0.072
District hospital	230	74.19	80	25.81		0.90	0.60	1.35	0.615
Primary clinic	1273	73.72	454	26.29		0.85	0.61	1.20	0.361
**Ownership of the hospital**					0.033				
Public hospital	521	77.18	154	22.82					
Non-public hospital	1610	72.98	596	27.01		1.50	1.15	1.96	0.003*

*Non-advanced: Stage 0, A, B; Advanced: Stage C, D. ^a^Chi-squared test. ^b^Event is an advanced stage of liver cancer; OR: odds ratio. ^c^Confidence interval. ^d^Logistic regression analysis. ^e^NT$: New Taiwan dollar. *P < 0.05

**Fig. 2:**
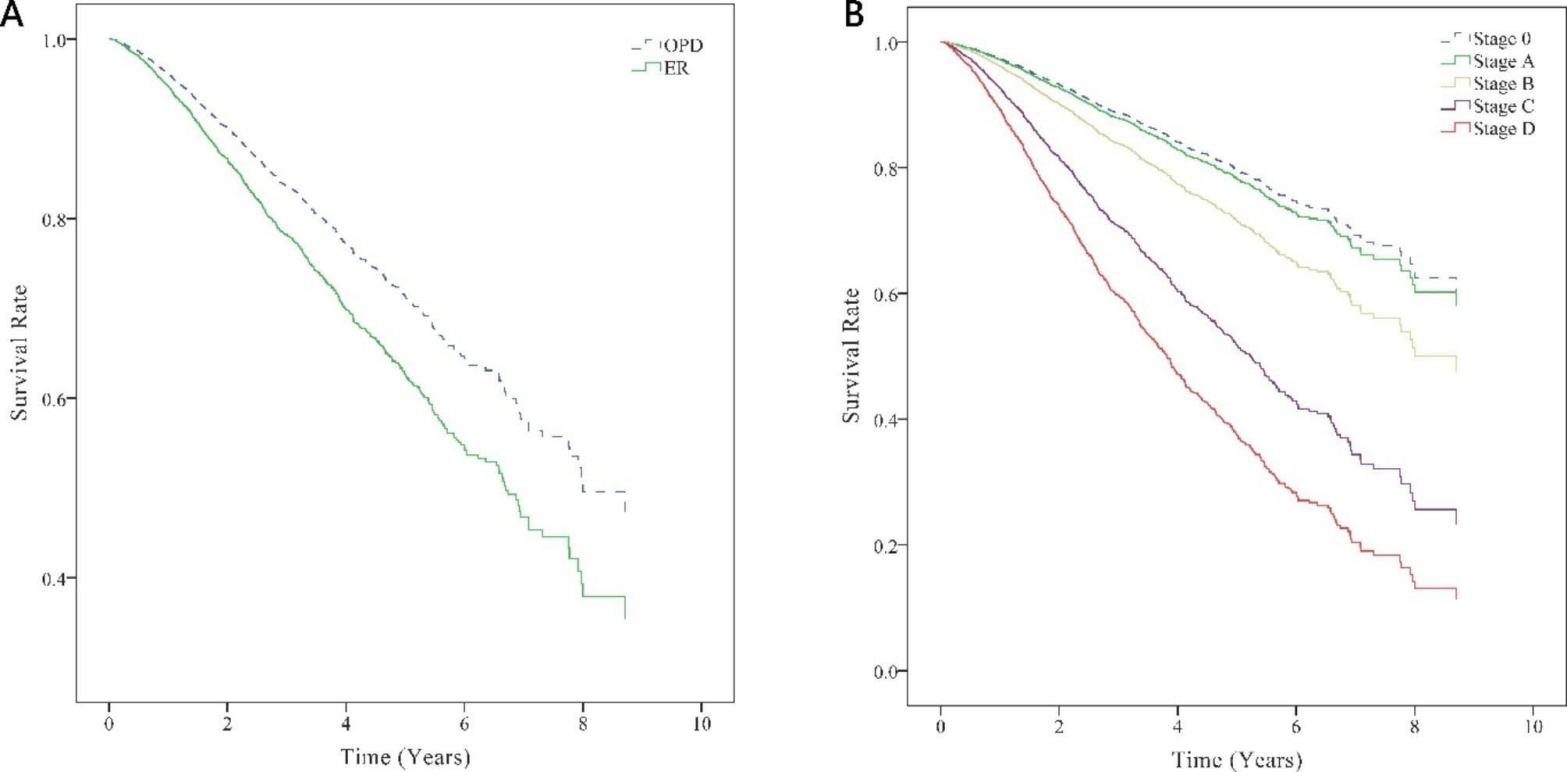
Survival curves of liver cancer-specific mortality (A) Survival curves of patients with first-ever liver cancer in the different locations of diagnosis (outpatient department [OPD] or emergency department [ED]). (B) Survival curves of patients with liver cancer in different stages at diagnosis

Table 4 presents the results of the Cox proportional hazard model used to explore the risk of liver cancer-specific death in the patients and related factors. Significant differences were observed based on the location of diagnosis, cancer stage, sex, monthly salary, education level, degree of urbanization of the residential area, use of preventive health services, and severity of comorbidities (*P* < 0.05). After adjustment for the study variables, the risk of death in the ED group patients was greater than that in the OPD group patients (aHR = 1.38, 95% CI = 1.14–1.68, *P* = 0.001). An association between initial ED diagnosis and poorer survival remains even after adjustment for the cancer stage at the time of diagnosis. Furthermore, Fig. 2 depicts the survival curves of patients who received an initial liver cancer diagnosis in an OPD versus those who received such a diagnosis in an ED after controlling for related variables and the survival curves of liver cancer patients at each cancer stage (Fig. 2). The patients with a higher monthly salary had a lower risk of death, with patients who received a monthly salary of NT$22,801–28,800 and > 28,800 exhibiting aHRs of 0.01 (95% CI = 0.01–0.03) and 0.03 (95% CI = 0.01–0.05), respectively. The patients with a higher education level also had a lower risk of death, with those having an education level of junior high school and senior high school (above) registering aHRs of 0.82 (95% CI = 0.70–0.97) and 0.78 (95% CI = 0.67–0.91), respectively. As for the residential area, the risk of death among the patients residing in places with level 4–7 urbanization was higher than that of those in level 1 areas (aHR = 1.27–1.72). The risk of death in patients who completed a health examination within 3 years prior to initial cancer diagnosis was 0.64 times (95% CI = 0.57–0.72) that of those who had not undergone such an examination. In terms of the severity of comorbidities, patients with liver cancer and a CCI score of ≥ 8 had a higher risk of death (aHR = 1.45, 95% CI = 1.06–1.98, *P* < 0.001) than those with a CCI score < 8.

**Table 4:** Variables associated with risk of liver cancer-specific mortality in first-ever diagnosed liver cancers

Variables	Censored		Death		P-Value ^a^	Adjusted HR	95%CI^b^		P-Value^c^
	N	%	N	%					
**Total**	1606	55.74	1275	44.26					
**Location of diagnosis**					< 0.001				
Outpatient department	1545	57.14	1159	42.86					
Emergency department	61	34.46	116	65.54		1.38	1.14	1.68	0.001*
**Stage**					< 0.001				
0	183	84.72	33	15.28					
A	844	70.86	347	29.14		1.08	0.75	1.55	0.675
B	364	50.28	360	49.72		1.47	1.02	2.13	0.040*
C	192	29.45	460	70.55		2.90	2.00	4.20	< 0.001*
D	23	23.47	75	76.53		4.32	2.81	6.66	< 0.001*
**Personal characteristics**									
**Gender**					0.020				
Male	1118	54.78	923	45.22					
Female	488	58.10	352	41.90		0.87	0.75	1.00	0.046*
**Age (year)**					< 0.001				
40–54	298	54.48	249	45.52					
55–64	522	58.52	370	41.48		0.85	0.71	1.02	0.072
65–74	489	56.14	382	43.86		0.82	0.68	0.98	0.034*
≥75	297	52.01	274	47.99		0.84	0.69	1.04	0.105
Mean ± SD	64.24 ± 10.48		64.85 ± 11.31		0.136				
**Marital status**					0.001				
Unmarried	76	49.03	79	50.97					
Married	1342	56.96	1014	43.04		1.14	0.90	1.45	0.283
Divorced	73	50.69	71	49.31		1.23	0.88	1.72	0.222
Widowed	115	50.88	111	49.12		1.07	0.78	1.46	0.688
**Socio-economic status**									
**Monthly salaries (NT$** ^**e**^ **)**					< 0.001				
≤ 22,800	533	29.76	1258	70.24					
22,801 − 28,800	651	98.64	9	1.36		0.01	0.01	0.03	< 0.001*
≥ 28,801	422	98.14	8	1.86		0.03	0.01	0.05	< 0.001*
**Education level**					< 0.001				
Elementary school (and illiterate)	813	52.72	729	47.28					
Junior high school	293	55.70	233	44.30		0.82	0.70	0.97	0.023*
Senior high school (and above)	500	61.50	313	38.50		0.78	0.67	0.91	0.002*
Environmental factors									
**Urbanization**					0.430				
Level 1	362	55.10	295	44.90					
Level 2	471	56.14	368	43.86		0.96	0.82	1.13	0.611
Level 3	246	55.41	198	44.59		1.01	0.84	1.22	0.940
Level 4	298	58.89	208	41.11		1.27	1.05	1.53	0.012*
Level 5	55	53.40	48	46.60		1.72	1.25	2.37	< 0.001*
Level 6	101	53.16	89	46.84		1.24	0.97	1.60	0.088
Level 7	73	51.41	69	48.59		1.39	1.06	1.82	0.019*
**Use of preventive medicine**									
**Health examination within three years before the diagnosis**					< 0.001				
No	267	33.67	526	66.33					
Yes	1339	64.13	749	35.87		0.64	0.57	0.72	< 0.001*
Health status									
**Charlson comorbidity index**					< 0.001				
≤ 2	327	87.20	48	12.80					
3–7	1075	56.37	832	43.63		1.32	0.98	1.79	0.068
≥ 8	204	34.06	395	65.94		1.45	1.06	1.98	0.020*
**Hepatitis B**					< 0.001				
No	934	52.83	834	47.17					
Yes	672	60.38	441	39.62		1.02	0.90	1.15	0.800
**Hepatitis C**					0.012				
No	1009	54.75	834	45.25					
Yes	597	57.51	441	42.49		1.01	0.89	1.15	0.848
**Characteristics of main treatment institutions after diagnosis**									
**Level of medical institutions**					< 0.001				
Medical center	244	49.39	250	50.61					
Regional hospital	416	52.46	377	47.54		1.02	0.86	1.21	0.807
District hospital	183	55.12	149	44.88		0.91	0.74	1.12	0.370
Primary clinic	763	60.46	499	39.54		0.83	0.71	0.98	0.030*
**Ownership of the hospital**					0.371				
Public hospital	242	54.50	202	45.50					
Non-public hospital	1364	55.97	1073	44.03		1.04	0.88	1.22	0.664

^a^Log-Rank test. ^b^CI: confidence interval. ^c^Cox Proportional Hazard Model. ^d^NT$: New Taiwan Dollar. *P < 0.05

## Discussion

The results of this study revealed that most of the liver cancer cases initially diagnosed in OPDs were stage A (42.53%), whereas most of those diagnosed in EDs were stage C (36.72%). Having stage C or D cancer, low monthly salary, CCI score ≥ 8, being divorced, not having hepatitis B, and attending a non-public hospital as the primary care institution before diagnosis were risk factors for initial ED diagnosis (*P* < 0.05, Table 1). Studies have confirmed that patients who received ED-based liver cancer diagnoses are more likely to be men, have advanced-staged cancer, and exhibit a higher incidence of large tumors and metastatic diseases that also yield symptoms such as decompensated liver cirrhosis, ascites, varicose bleeding, abdominal pain, and weight loss [8]. One study has also shown that in OPDs, the progression from liver disease to liver cancer can be monitored every year, and the detection rate is 1.6% [20]. Another study revealed that half of the local lesions detected by outpatient ultrasound were liver cancer cases and most of the patients received diagnoses of BCLC stage 0 or A cancer [21]. Therefore, cancer cases diagnosed in an OPD are mostly detected at an early stage, and the results of our study also indicate that the proportion of early-staged cancer diagnoses (stages 0, A, and B) was higher in OPDs than in EDs (75.26% vs. 54.23%). The aOR of developing advanced-stage cancer was 1.77 times higher in the ED group than in the OPD group (Table 3), which accords with the results of [8].

Although an ideal liver cancer diagnosis policy would involve screening patients with risk factors in outpatient clinics, because most high-risk patients have limited access to medical services, many US citizens (32%) receive an initial liver cancer diagnosis in an ED [8], where the proportion of uninsured individuals is higher. Our research revealed that Taiwan’s proportion of initial liver cancer diagnoses in EDs (6.14%) is lower than that of the United States. This may be due to Taiwan’s implementation of the NHI scheme. In the United Kingdom, which has a universal healthcare system, cancers of different organs are initially diagnosed in EDs in 13.9–29% of cases, but no case of liver cancer was initially detected in an ED in two British studies [22, 23]. This may be because of the low prevalence of liver cancer in the United Kingdom (approximately a third of that in Taiwan or Eastern Asia) [24] or because most patients with liver cancer have been screened out in the OPD. Therefore, compared with the United Kingdom, Taiwan still has room for improvement in outpatient screening and the prevention of liver cancer. In South Korea, the National Cancer Screening Program provides free liver cancer screening services twice a year for the low-income insured (bottom 50%) [25]. Although no studies yet have shown that the program has the effect of reducing liver cancers diagnosed in the ED, it is still a good policy that the Taiwan government can emulate.

The present study demonstrates that advanced stage liver cancer (aOR = 0.76, 95% CI = 0.62–0.92, *P* = 0.005; Table 3) and death (OR = 0.64, 95% CI = 0.57–0.72, *P* < 0.001; Table 4) were less likely among those who received adult health examinations. However, the use of health examination did not exert a significant effect on the likelihood of liver cancer diagnosis in an ED (aOR = 0.86, 95% CI = 0.61–1.21, *P* = 0.381; Table 1). At present, the adult preventive health care service subsidized by the Taiwan government provides a free health exam every 3 years for people aged over 40 and under 65 years. Moreover, indigenous people aged over 55 years, people with poliomyelitis sequelae aged over 35 years, and people aged over 65 years are entitled to one free health exam every year. The examination items include basic questionnaire surveys (inquiring into disease and family history), physical examinations (general physical examinations, blood pressure, and body mass index), laboratory examinations (urinalysis and basic blood biochemical examinations), and health consultations (regarding quitting alcohol and betel nut consumption and maintaining a healthy diet and weight) [26]. People born after 1966 and aged over 45 years or indigenous people aged over 40 years can receive a free hepatitis B and C screening once in their lives [27]. Abdominal ultrasonography and α-fetoprotein analysis, which are recommended for liver cancer detection [6], are not provided in these adult preventive health examinations or hepatitis B and C screening services. Regarding cancer screening, the government currently provides screening services for four cancers, namely cervical, breast, oral, and colorectal cancers [17]. Liver cancer screening is not included in the cancer screening service in Taiwan.

Our results also revealed that most liver cancer patients with hepatitis B and C receive cancer diagnoses at a non-advanced stage, which was similar to the findings of a previous Japanese study [28]. The reason may be that people with hepatitis B and C have regular follow-up treatments, and liver cancer is therefore largely detected and controlled early. In a Swiss study, all of the participating patients with liver cancer received a diagnosis at an early stage through ultrasound examination in hepatology outpatient clinics [21]. For the years spanning 2012 to 2015, the numbers of people in Taiwan who were screened for hepatitis B and C were 38,000, 57,000, 76,000, and 89,000, respectively; the positive rates of hepatitis B and C screening were approximately 16.0–16.3%, and 3.8–4.7%, respectively, and these rates have continued to increase [29]. In a sample survey of 4,928 people aged 25–69 years, 69.1% had been tested for hepatitis B and C, and 12% received diagnoses of hepatitis B or C after being screened. After becoming aware they had hepatitis B or C, 69% of people sought medical treatment. As of 2017, a total of 476,341 people had received hepatitis C screening services in Taiwan, and the average hepatitis C positive screening rate was 4.3% [30]. Therefore, a considerable proportion of people have still not received hepatitis B and C screening or have not received further treatment after receiving hepatitis B or C diagnoses.

Studies have found that married patients are less likely to have advanced tumors. Social support from the spouses was associated with lower cortisol levels and higher natural-killer cells which may result in decreasing tumor progression. The spouses may also encourage patients to receive definitive versus expectant management [31, 32]. Our study results also indicated that the probability of receiving an advanced-staged cancer diagnosis is lower in married patients than in unmarried people, although the difference is non-significant (aOR = 0.69, 95% CI = 0.47–1.01, *P* = 0.055). Our study results also revealed that the higher the monthly salary, the lower the probability of receiving a late-stage cancer diagnosis (Table 3), which is consistent with the results of previous studies [33].

According to the results of this study, the risk of liver cancer-specific death was 1.38 times higher in the ED group than in the OPD group (95% CI = 1.14–1.68, *P* < 0.001). ED diagnosis of liver cancer is associated with poorer survival even after adjustment of other variables (Table 4). The survival curves of the OPD and ED groups (Fig. 2) also indicate that the 1-, 3-, and 5-year survival rates were lower in the ED group than in the OPD group. A South Korean study reported that liver cancer is one of the most common cancers in the ED and the in-hospital mortality was 19.7% [34]. Another South Korean study reported that 24% of patients with liver cancer that ruptured in the ED died within 30 days and 50.4% died within 90 days [35], which also demonstrated a similar poor outcome in the ED.

This study’s results suggest that compared with patients with stage 0 liver cancer, the risk of death for patients with stage B, C, and D liver cancer were 1.47, 2.90, and 4.32 times higher, respectively. This indicates that the more advanced the liver cancer is, the higher the risk of death (Table 4), a finding consistent with the results of previous studies [36, 37]. The results of our study reveal that women with liver cancer have a significantly lower risk of death than men (aHR = 0.87, 95% CI = 0.75–1.00, *P* = 0.046; Table 4). This finding is consistent with the results of previous studies. Female sex has a protective effect on liver cancer progression [38]. One study revealed that the prognosis of women with liver cancer is superior to that of men, and women tend to be diagnosed at an older age and an earlier stage (stage 0 or A) [39]. In our study, the risk of death among patients in level 4 to level 7 (least urbanized) residential areas was higher than that among patients in highly urbanized cities (level 1), with aHRs of 1.27–1.72. Studies have shown that the risk of liver cancer mortality among rural residents is greater than that of urban residents [33, 40]. This distinction may relate to medical care and resource disparities between urban and rural areas. The relative survival rate of urban residents is reportedly over twice that of rural residents [40], which is consistent with our findings. Our study of comorbidity revealed that the higher the severity of the comorbidity, the higher the risk of death. A previous study reported that compared with patients with a CCI score of ≤ 5, the risk of death in patients with scores of 6 and ≥ 7 were 1.5 and 2.5 times greater, respectively [41]. Our study results suggest that the patients with CCI scores of ≥ 8 had a higher risk of death than those with lower scores (aHR = 1.45, 95% CI = 1.06–1.98, *P* = 0.020).

## Limitations

To our knowledge, this is the first cohort study to compare ED and OPD-diagnosed liver cancers in Taiwan, which has comprehensive population coverage by NHI (coverage rate of 99.7%) [42]. A similar existing study was conducted in the US with only 55% population having government insurance. The strengths of our study are the population-based design, long study period, and large sample size. However, several limitations must be noted. Because the NHIRD was used for analysis in this study, only the reimbursement data could be presented, and it was impossible to know whether patients undertook self-paid health examinations. In addition, miss-coding of the liver cancer ICD codes in the ED may also have consequences for the underestimation of liver cancer patients in the ED. Finally, patients may have visited an ED for temporary treatment but then been referred to receive further confirmatory examinations in the OPD, which may have resulted in an underestimation of the number of liver cancer cases diagnosed in EDs and an overestimation of those diagnosed in OPDs.

## Conclusion

This study suggests that liver cancer cases initially diagnosed in EDs tend to be more advanced in stage than those diagnosed in OPDs (45.76% vs. 24.74%). Although patients who have received prior adult health examination are less likely to receive advanced stage cancer diagnoses (aOR = 0.76, 95% CI = 0.62–0.92, *P* = 0.005; Table 3) or die from cancer (aOR = 0.62, 95% CI = 0.56–0.69, *P* < 0.001; Table 4), the current Taiwanese health examination service (without liver cancer screening) is insufficient to ensure that cancer diagnoses are received in OPDs rather than EDs (*P* = 0.381). The patients who receive initial liver cancer diagnosis in EDs are at a higher risk of advanced stage cancer (aOR = 1.77, 95% CI = 1.27–2.47, *P* < 0.001) and death (aHR = 1.38, 95% CI = 1.14–1.68, *P* = 0.001) even after adjustment of other related variables. The government may consider further implementing liver cancer screening for high-risk and low-socioeconomic people for early detection and treatment.

## Data Availability

In this study, three databases were used, namely the NHIRD, the Taiwan Cancer Registry, and the Cause of Death File. Data are available from the Ministry of Health and Welfare, Taiwan. Due to legal restrictions imposed by the Taiwanese government related to the Personal Information Protection Act, we cannot make these databases publicly available. Researchers can apply for access to use them in their studies. Requests for data can be sent as a formal proposal to the Science Centre of the Ministry of Health and Welfare (https://www.mohw.gov.tw/mp-2.html). Removing raw data from the Science Centre is not permitted. Only the analytic outputs in the form of tables or figures can be printed out.

## List of Abbreviations

ED: Emergency Department
OPD: Outpatient departments
CI: Confidence interval
NT$: New Taiwan Dollar
BCLC: Barcelona Clinic Liver Cancer
NHIRD: National Health Insurance Research Database
ICD: International Classification of Diseases
ICD-O-3: International Classification of Diseases for Oncology, third edition
CCI: Charlson comorbidity index
aHR: Adjusted hazard ratio
aOR: Adjusted odds ratio
